# Umbilical granuloma frequency of newborns in Third-line Hospital in Turkey

**DOI:** 10.4314/ahs.v22i2.64

**Published:** 2022-06

**Authors:** Öztaş Tülin, Asena Muhammet

**Affiliations:** University of Health Sciences GaziYaşargil Training and Research Hospital, Department of Pediatric Surgery, Diyarbakir, Turkey

**Keywords:** Umbilical granuloma, umbilical cord seperation, newborn bath, umbilical granuloma frequency

## Abstract

**Background/Objectives:**

The aim is to determine the umbilical granuloma frequency of newborns and etiological factors.

**Methods:**

In this study, the records of 21344 newborns who were admitted to our hospital between February 2015 and August 2019, were examined.

**Results:**

21191 newborns are included in the study. 2.4% of newborns was Syrian refugee and others were citizens of Turkey. Umbilical granuloma frequency was % 3.83. While umbilical granuloma frequency was 3.85% in Turkish citizen newborns, %3.01 in Syrians. Mean umbilical cord seperation time was 7.1 days in cases with umbilical granuloma. There was no statistically significant relationship determined between umbilical granuloma development and race and time of umbilical cord seperation (p >0.05) The frequency of umbilical granuloma was 3.5% for boys and 4.1% for girls. Umbilical granuloma was being observed statistically significantly higher in girls than in boys (p <0.05). 80.8% of the cases with umbilical granuloma were bathed before the umbilical cord seperation. A significant difference was determined between bathing before umbilical cord seperation and umbilical granuloma development (p < 0.05).

**Conclusion:**

Umbilical granuloma, with frequency of 3.83% in newborns. Umbilical granuloma is more common in girls and newborns bathed before the umbilical cord seperation.

## Introduction

Umbilical granuloma (UG) is the most common benign umbilical pathology in newborns 1–6. UG is seen as a small red colored flesh which has rarely bloody discharge that makes stain on underwear after cord seperation^7^. Usually in the first two weeks after birth, the cord separates and a fine granulation tissue forms on the umbilicus surface. One or two weeks after the cord separates, the granulation tissue disappears with epithelization of the umbilical cord and the umbilicus is covered with normal skin. However, if the umbilical cord is not sufficiently epithelized, the granulation tissue turns into granuloma.^3^–^5^,^8^. Differential diagnosis of UG with diseases such as vitelline canal or omphalomesenteric canal residue, umbilical polyp, umbilical mass shall be made^9^.

The etiology of UG is not fully known, but the mode of delivery, umbilical care method, moist in umbilicus, infection, delayed cord separation are the factors that are thought to be related with UG 9,10. Clean and dry cord care recommended by the World Health Organization (WHO) is being implemented in our hospital. With dry cord care, the risk of infection in the cord is reduced and the umbilical cord separates earlier^11^. Topical antiseptic application has been recommended by WHO only in poor hygienic conditions and severe infections^12^. Babies born in our hospital are discharged after being kept under observation for 24 hours. Before the mothers are discharged, our health care personnel recommend them to keep the cord dry, not to take a bath before the umbilical cord separates and to nappy the diaper under the cord. During discharge, families are provided with information form including cord care. Newborns are called to the pediatric outpatient clinic for routine control on the 5th and 30th days after being discharged. In our region, families bathe their babies for cleaning purposes due to their own cultural beliefs and when the belly is contaminated with urine or stool, before the cord separates.

UG has been reported to occur in one in 500 newborns^7^,^13^,^14^. But UG is observed much more common. There are very few studies about the frequency of UG in the current literature. It is important to know the etiological factors to both reduce the frequency of UG and expenses on health. The aim of this study is to determine the frequency of UG and related etiological factors in newborns.

## Materials and Methods

The study was conducted in the pediatric surgery clinic of our hospital between October 2019 and January 2020. In this cross-sectional epidemiological study, the demographic data of 21344 newborns who applied to the pediatric and pediatric surgery outpatient clinic of Health Sciences University Diyarbakır GaziYaşargil Training and Research Hospital between February 2015 and August 2019 were analyzed retrospectively.

Newborns whose mothers have systemic disease, hepatitis or genital infection or have premature membrane rupture, or born with using vacuum or forceps, and those with cardiac or vascular anomalies and immune system pathology were not included in the study.

Examination record forms composed of diagnosis, complaints, history, self-history, family history, examination, suggestion and treatment sections are being used for each patient who comes to the outpatient clinic in our hospital. Such forms are filled in by the doctor who examined the patient and recorded in the database of hospital. The examination record forms of the newborns who came to the pediatric and pediatric surgery outpatient clinic for postnatal (5–30 days) control were examined. Age, gender and nationality information are recorded in the examination registration forms. During examination, pediatric or pediatric surgeon questions mothers on the type of delivery, cord care, time of umbilical separation and whether babies had bath before the umbilical cord separates. Furthermore, it is recorded whether there is an umbilical infection and granuloma during physical examination. Newborns were divided into two categories as with and without UG. The families of the newborns whose examination record forms were missing, were reached by phone by two physicians (one of them an interpreter), independent of the study.

In the forms prepared for the study, the age, gender, nationality, mode of delivery, umbilical care, time of umbilical cord separation, and whether babies had bath before the umbilical cord separated were recorded.

This study was approved by the Clinical Research Ethics Committee of Health Sciences Diyarbakır GaziYaşargil Training and Research Hospital University (27.09.2019/Number: 332).

### Statistical methods

Data obtained in the study were statistically analyzed using SPSS Statistics for Windows, Version 22.0. (IBM Corp. Released 2013. Armonk, NY). Categorical variables were expressed as numbers (n) and percentages (%) and the Kolmogorov-Smirnov test was used to examine the normal distribution of continuous data. Numerical variables with normal distribution were shown as mean ± standard deviation. Normally distributed numerical variables were compared by Student's T test. Pearson's Chi-Square Test was used to determine whether there were differences in categorical variables. In all data, p <0.05 was considered as statistically significant.

## Results

For the 21191 newborns included in the study, mean age was determined as 15.8 days and %2.4 of newborns were Syrian refugees while others were Turkish nationals. Of the total 21191 newborns, 53.8% were boys and the remaining 46.2% were girls. 32.9% of the newborns included in the study were born vaginally and 67.1% by cesarean section. Umbilical of all of the newborns who participated in our study were classically clamped and had no antibiotic pomade, disinfectant or anything else used for umbilical care. Dry cord care was applied. The mean time for separation of the umbilical cord was 7.4 ± 2.6 days (3–14 days) in babies born vaginally, while it was 8.1 ± 3.6 days (4–14 days) in babies born by cesarean section. In our study, no statistically significant relationship was determined between delivery type and umbilical cord separation time (p = 068).

UG was detected in a total of 813 cases, 50.5% of which were boys and 49.5% of them were girls with an average age of 5.6 days (7–30 days). In our study, the determined frequency of UG was 3.8% and this rate was 3.5% in boys and 4.1% in girls. UG was determined statistically significantly more common in girls than boys (P = 0.05). Of the newborns with UG, %98.1 were Turkish citizens while %1.8 were Syrian refugees. The frequency of UG in Turkish and Syrian newborns were 3.85% and 3.01%, respectively. In our study, considering UG prevalence, statistically significant relationship was not determined between Turkish citizens and Syrian refugees (p=0.34). 32.1% of the newborns with UG were born vaginally and 67.9% by cesarean. 33.1% of the newborns without UG were born vaginally and 66.9% by cesarean. In our study, there was no statistically significant relationship between UG development and delivery type (p = 0.58).

While the mean time of cord separation in newborns with UG was 7.1 days (5–10 days), it was 7.3 days (4–20 days) in newborns without UG. It was 7.09 days for Syrian refugees (6–10 days). There was no statistically significant relationship determined between the development of UG and the cord separation (p = 0.05). While 80.8% of the cases with UG were bathed before the umbilical fall, 74.5% of the newborns without UG were bathed. There was a significant difference between taking a bath before the cord separation and the development of UG (p = 0.001). The distribution of the sociodemographic data of the newborns included in the study is presented in Table 1.

**Table 1:** Demographic and clinical characteristics of newborns

Patients characteristics	Infant with UG	Infant without UG	P value
	n =813	n=20378	
	n(%)	n(%)	
Mean gestational age ±sd (weeks)	38.9 ±1.3 (38–40)	38.8±1.1 (38-0)	0.59
Mean weight ±sd (grams)	3,105.6 ±428.9	3100.8±546.8	0.57
Gender			0.04
Female	402(49.5)	9376(46.1)	
Male	411(50.5)	11002(53.9)	
Nationality			0.34
Turkey	798(98.2)	19895(97.6)	
Syrian	15(1.8)	483(2.4)	
Birth method	0.58		
Vaginal	261 (32.1)	6732(33.1)	
Cesarean	552 (67.9)	13646(66.9)	
Mean umbilical cord seperation time (day)	7.1(5–10)	7.3(4–20)	0.05
Bathed before the umbilical separation	657(80.8)	15182(74.5)	0.001

UG: Umbilical granuloma

## Discussion

According to the results of this study, the frequency of UG in newborns is 3.83%. To the best of our knowledge, there has not been any study investigating the frequency and etiology of UG in the current literature. Our study has the largest patient population evaluating UG frequency and associated factors.

Cord care method may be associated with granuloma development^10^. In a study conducted with 669 newborns, it was reported that UG developed with chlorhexidine in 12.8% after umbilical care and 11.7% after dry cord care^13^. It is stated that frequency of UG decreases with dry and clean cord care^10^. In studies on umbilical care, the frequency of UG has been reported at different rates (2.2%, 0.06%)^11^,^15^. Dry cord care was applied to 21191 newborns participating in our study and the frequency of UG was determined as 3.83%.

Although UG is very common in newborns, its etiology is still unknown. It has been reported that UG is seen at the same frequency in boys and girls regardless of gender 4,8. In one study, it was stated that it was seen more in boys, boys / girls ratio was 1.1/1, while another study indicated that the boys/girls ratio was 1.7/11,4. Contrary to previous studies, the girls/boys ratio in our study was determined as 1.1/1 and the frequency of UG was statstically significantly higher in girls.

There are various opinions on the relationship between delivery method and umbilical pathologies. It has been reported that the period of umbilical cord separation takes longer in newborns born by cesarean section^13^. As in our study, it was reported that there was no relationship between the mode of delivery and the time of umbilical cord separation^16^. To the best of our knowledge, there is no study in the literature conducting on the relationship between UG and delivery method. The results of our study reveal that the mode of delivery is not related to the time of separation of the umbilicus and the development of UG.

UG develops as a result of mild inflammation of non-epithelialized granulation tissue in the umbilicus^4^,^15^,^17^. Dry cord care reduces the risk of infection^10^. Due to the war in Syria, there has been a mass flow of immigrants to our country and therefore, a considerable number of Syrian children live in our country. Syrian refugees included in our study have low socioeconomic status and poor postnatal hygiene. In our study, the frequency of UG in Turkish newborns was determined as 3.85%, while this rate was 3.01% in Syrians. The results of our study suggest that umbilicus infection is very rare with dry cord care and infection has no role in the etiology of UG.

Separation time of the umbilical cord may be related to the development of UG 9 . The separation time of the umbilical cord varies depending on the race, geographic location and hygiene^18^. In the study, no difference was found in the time of umbilical cord separation in Turkish and Syrian newborns. As determined in our study, some studies suggest that ethnic origin and hygiene at birth do not affect the development of umbilical cord pathology 5,15. In case umbilical cord, which is normally expected to separate within 7–15 days after birth, separates in more than 3 weeks, then it is defined as delayed cord separation^19^. It is stated that the umbilical cord separation time is related to the umbilical care method and the cord separation time is shorter after dry cord care in comparison with other methods^16^. The results of our study are compatible with the literature, and no delayed cord separation was observed in patients after dry cord care. While it was reported in a study that delayed cord separation might play a role in UG development^9^, another study reported no relationship between cord separation time and UG 16 The results of our study support that the umbilical cord separation time has no role in the development of UG. Bathing before the umbilical cord separation is thought to be one of the factors that play a role in umbilical cord pathologies. Bathing in the neonatal period is not an absolute contraindication^20^. It is stated that bathing newborns is beneficial in terms of hygiene, but it is recommended not to bathe until the umbilicus separates since it is thought hypothermia may develops after bathing^20^. In the study conducted by Ayyıldız et al., it is stated that taking a bath causes cord to remain wet and late separation of umbilical cord and the umbilicus tends to get infected^10^. The results of our study suggest that in newborns who are bathed before the umbilical cord separation, the cord stays wet after bath, the epithelization of the cord is delayed, and therefore UG develops. In our study, bathing before cord separation was determined as a condition related to UG.

The strengths of our study are that, it is conducted with a large number of patients and it includes participants from different socioeconomic conditions and cultures. The limitation of our study is that it was conducted retrospectively.

Consequently, uncertainties in UG etiology continue. UG is more common in girls and newborns who were bathed before cord separation. UG, with frequency of 3.83% in newborns in living Turkey, remains the most common characteristic of being a benign umbilical pathology of the newborn. Dry method shall be preferred in core care. If there is no infection, it is not necessary to use an antiseptic for cleaning the cord. In case the cord is contaminated with urine or stool, it shall be cleaned with water and dried. Bathing newborn shall not be preferred before cord separation. Babies who take a bath before the cord separation shall be dried finely. Attention shall be paid not to keep the umbilicus moist. The frequency of UG may be reduced by very good drying of babies after bathing or bathing after umbilical separation.
